# Supplementary figures and images for: The PMA phorbol ester tumor promoter increases canonical Wnt signaling via macropinocytosis
Source: eLife. 2023 Oct 30;12:RP89141. doi: 10.7554/eLife.89141 (PMC10615368; doi:10.7554/eLife.89141)

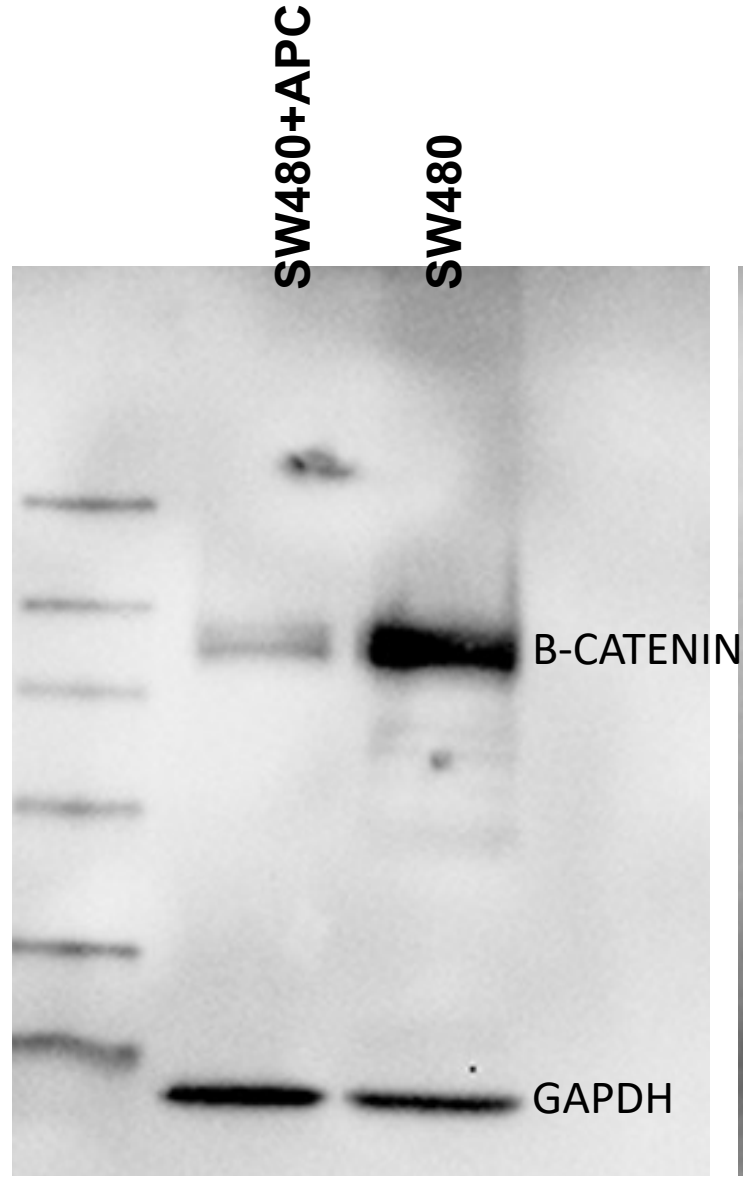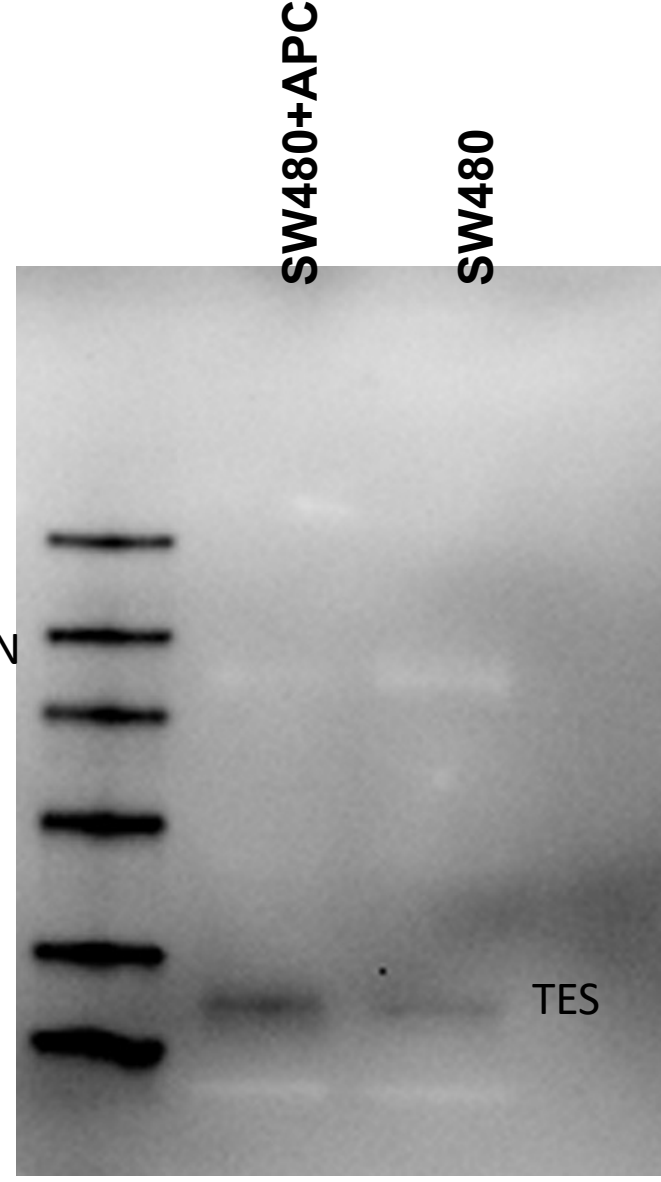

Supplement: Figure 6—source data 1. [file elife-89141-fig6-data1.zip › Figure 6-Source data 1.pdf]
